# Recovery of Polyphenols from Vineyard Pruning Wastes—Shoots and Cane of Hybrid Grapevine (*Vitis* sp.) Cultivars

**DOI:** 10.3390/antiox10071059

**Published:** 2021-06-30

**Authors:** Reelika Rätsep, Kadri Karp, Mariana Maante-Kuljus, Alar Aluvee, Hedi Kaldmäe, Rajeev Bhat

**Affiliations:** 1 ERA-Chair for Food (By-) Products Valorisation Technologies (VALORTECH), Estonian University of Life Sciences, 51006 Tartu, Estonia; rajeev.bhat@emu.ee; 2 Polli Horticultural Research Centre, Institute of Agricultural and Environmental Sciences, Uus 2, 69108 Polli, Estonia; Alar.Aluvee@emu.ee (A.A.); hedi.kaldmae@emu.ee (H.K.); 3 Institute of Agricultural and Environmental Sciences, Estonian University of Life Sciences, Fr. R. Kreutzwaldi 1, 51006 Tartu, Estonia; kadri.karp@emu.ee (K.K.); mariana.maante-kuljus@emu.ee (M.M.-K.)

**Keywords:** grapevine polyphenols, stilbenoids, vineyard waste, sustainable viticulture, valorization

## Abstract

Grapevine shoots and canes represent a significant amount of biomass, considered as a waste in viticulture. In cooler climates, grapevines are pruned in the autumn (October) and spring (March) due to harsh winter conditions (e.g., snow, low temperatures), and large amounts of biomass are produced at these different pruning times. This work was undertaken in order to investigate the potential of vineyard pruning waste for recovery of polyphenolic compounds for biomass valorization. Qualitative and quantitative analyses of grapevine shoot and cane polyphenols, including flavonoids and stilbenoids were performed using UHPLC MS/MS method. The results revealed the flavonols (quercetin) to be the most abundant compounds in shoots among all the three cultivars screened (Zilga, Hasansky Sladky, Rondo). Stilbenoids (ε-viniferin) dominated in the canes, while increased level of flavonols with lower contents of stilbenoids was detected in the endo-dormant canes, and higher amounts of flavanols and stilbenoids were recorded in eco-dormant canes. In conclusion, the content of polyphenols in grapevine shoots and canes differed among the cultivars and dormancy phases. The results generated from the present study contribute to the sustainable and environmentally friendly viticulture practice via valorization of vineyard pruning wastes.

## 1. Introduction

In the European Union, area under grapevine cultivation is between 3–4 million hectares [1]. In Estonia, viticulture is a new trend in horticulture, but is a fast-growing field of interest to the local farming community. The production residues from vineyard occur in remarkable amounts as vine shoots and canes. Valorization of such wastes can be an option as a potential source of bioactive components that could be of interest in the field of pharmaceuticals, cosmetics, food industry. Additionally, effective valorization can also contribute to the sustainability and decreased environmental effects in viticulture [2,3,4,5]. Grapevine wastes and by-products, specifically herbaceous shoot and woody cane material, are the major leftovers of vineyard management (pruning, trimming) and constitute a considerable amount of rejected biomass. 

There are more than 183 polyphenolic compounds that have been identified in the roots, woods, canes, stems, and leaves, mainly including 78 stilbenoids, 15 hydroxycinnamic acids, 8 flavanones, 35 flavonols and many other groups of compounds [6]. Moreover, grapevine pruning wastes such as canes and shoots have a high potential for valorization by recovery of remarkable amount of natural bioactive phytochemicals [7,8,9,10,11,12,13]. Vine-shoots and canes accumulate various non-volatile (flavonols, phenolic acids, stilbenoids etc.) and volatile phenolic compounds (alcohols, benzenoids, esthers, furanics, lactones, terpenes etc.) [8,14,15,16]. Grapevine canes’ stilbenoid concentration and composition depends on *Vitis* species and cultivar [14,17,18], as well as on the conditions of cultivation (plant management, climate conditions etc.) [13,14,19,20]. In the plant’s stilbenes participate in constitutive and inducible defense mechanisms and play a role in plant–pathogen and plant–herbivore relationships, and in plants subjected to abiotic stress conditions [14,19,21,22]. Moreover, mechanical wounding of freshly pruned canes induced the stilbenoid metabolism in the grape canes (E-resveratrol and E-piceatannol) [9]. According to Guerrero et al. [14], one-year-old canes of grapevines contained ten major stilbenoids, but the most abundant was ε-viniferin (26–52%), while trans-resveratrol and piceatannol varied the most with the years. Total stilbene concentration ranged 2400–5800 mg kg^−1^ d.w. [14]. Cebrián et al. [8] have demonstrated that in two different *Vitis vinifera* cultivars, the vine-shoots have accumulated various phenolic compounds after 1, 3 and 6 months of post-pruning storage. In particular, long-term storage effects on stilbenoid concentration (i.e., trans-resveratrol and trans-ε-viniferin) in canes and grape cluster stems during ripening of grapes was investigated, but no clear trend was observed [15]. The contents in canes ranged 441–7532 and 1218–5341 mg kg^−1^ (d.w.) for trans-resveratrol and trans-ε-viniferin, respectively, depending on cultivar, vintage and storage time [15]. Grapevine canes are described as a promising source for obtaining cane stilbene enriched extracts with antifungal properties, but as well due to their high antioxidant activity suggested as a good raw material for nutraceutical applications [14]. Grapevine-shoot extract with high concentration on stilbenoids (29%) has been used in wine matrix as a promising alternative in order to reduce, but not to replace SO_2_ in white wines [11]. Toasted grapevine canes contained interestingly considerable amount of pro-delphinidins that together with the stilbenes may favor antioxidant activity of wine [10]. Leaf and cane extracts possess valuable antioxidants and other biochemical compounds that could be useful for production of nutraceuticals and pharmaceuticals in terms of human consumption as well [23].

Most of the research has been done on leaves, bunch stems and canes of *V. vinifera* L. cultivars [4,13,14,18,20,24] or even on some wild *Vitis* genotypes [25], but less information is available on the hybrid grapevine cultivars [17,23,26], which are more suitable for growing in cool climate conditions due to their cold-hardiness. As grapevines enter endo-dormancy with leaf fall and after required chilling units the eco-dormancy driven by low temperatures follows, both dormancy phases have significant impact on the vine cane biochemical composition [27]. Some of the earlier publications on biochemical composition of vegetative plant organs of hybrid grapevine cultivars showed that the canes cultivated in cool climate conditions in Estonia could be an excellent source of dietary stilbenoids, resveratrol, and viniferin [17,23]. However, there is still a scarcity of knowledge about the effect of dormancy phases on the bioactive compounds in woody canes of hybrid grapevine cultivars in cool climate conditions.

Based on this, the aim of the present research was to determine the content of polyphenols and some of the major individual polyphenolic compounds in grapevine shoots and canes, based on their pruning time and plants’ dormancy phase. Moreover, as the extraction method and conditions can significantly affect the final recovery of the beneficial compounds [28,29], in the present study we selected microwave assisted extraction, which is also considered as a green extraction technique. It is expected that the results generated in the present study will immensely contribute to the sustainable and environmentally friendly viticulture practices.

## 2. Materials and Methods

### 2.1. Experimental Site and Plant Material

The experimental vineyard at the Estonian University of Life Sciences experimental station in Tartu County (58°21′ N; 26°31′ E) was established in 2007 with own-rooted plants of hybrid grape cultivars. Vines planting density was with 2 × 2 m spaces, trained on low double trunk trellis with 12 buds left per plant. The vine rows were North-to-South oriented; woven ground cover fabric was used in rows; no irrigation was used and no fertilizers were added. Experimental cultivars: 

‘Hasansky Sladky’ (synonyms: ‘Hasan Sweet’, ‘Varajane Sinine’, ‘Baltica’) is a vigorous Russian early ripening hybrid grape cultivar for wine. The pedigree includes *Vitis amurensis* L. and *Vitis labrusca* L. (Ruprecht × ‘Dalnevostochnyi Tikhonova’). The vines are quite disease resistant to grape downy mildew (*Plasmopara viticola*) and have good winter hardiness.

‘Zilga’ is a Latvian early ripening hybrid cultivar for wine. The pedigree includes *Vitis amurensis* L. and *Vitis labrusca* L. [(‘Smuglyanka’ × ‘Dvietes’) × ‘Jubileinaja Novgoroda’)]. The vines have a very vigorous growth habit and high yield.

‘Rondo’ is a German medium ripening hybrid grapevine cultivar for red wine. The pedigree includes *Vitis amurensis* L. (‘Zarya Severa’ × ‘Saint Laurent’). The growth habit of vines is vigorous, but the canes do not mature on time and therefore suffer from cold and winter damage in Estonian cool climate conditions.

The experimental area in Tartu County belongs to a very cool vine-cultivating zone as per the heliothermal index [30]. The average length of the frost-free period was 158 days and the first autumn frost was in the second half of October. Compared to an average of 30-years, February, March, April and June in 2019 were much warmer as compared to all of the winter months in 2019/2020 (see Table 1). Majority of the plants entered the endo-dormancy on time in October 2019 with the arrival of autumn weather conditions. Further, no winter damage of grapevines was recorded during the year 2019/2020.

### 2.2. Collection of Shoots and Canes and Preparation of Samples

The grapevine shoots and canes were collected based on the cultivars and the most common pruning times used for vineyard management in Estonia. The vine shoots were pruned in July 2019, and canes in the phase of endo-dormancy in October 2019, and in eco-dormancy in March 2020. The samples (approximately 2 kg) were collected randomly from the total amount of pruned shoots and canes and pooled for obtaining an average of a sample. The fresh samples were stored at −20 °C until further use. 

Grapevine shoots and canes were dried during 24 h at +40 °C using Zelmer circular-air dryer (Zelmer 300W, SDA Factory Vitoria, Vitoria-Gasteiz, Spain) and then ground with Retsch cutting mill (Retsch SM 300, Retsch GmbH, Haan, Germany) to obtain a homogenous sample (diameter of sieve holes was 2 mm). The dry weight of each sample was determined using Precisa moisture analyzer (EM 120-HR, Precisa Gravimetrics AG, Dietikon, Switzerland). The extraction of the shoot and cane powder was performed in triplicates as follows: for the recovery of the polyphenolic compounds, approximately 2 g of dried ground powder was weighed into the 200 mL beaker and 100 mL of 60% ethanol-water solution (*v*/*v*) added. The mixture was treated using microwave-assisted extraction (MAE) method, extraction time was 5 min and power 100 W (NEOS GR Microwave Extraction System, Milestone Inc., Shelton, CT, USA). Extraction parameters (time, power and solvent ratio) were chosen and slightly modified according to Piñeiro et al. [28] and Jesus et al. [29]. After the processing, the extracts were filtered through Whatman filter paper no 1 and stored at −20 °C until the analyses of individual polyphenolic compounds and total phenolic content (TPC).

### 2.3. Identification and Quantification of Polyphenols by LC-MS Method

Qualitative and quantitative analyses were performed as described by Ben-Othman et al. [31], on a Shimadzu Nexera X2 UHPLC with mass spectrometer LCMS 8040 (Shimadzu Scientific Instruments, Kyoto, Japan). The UHPLC system was equipped with a binary solvent delivery pump LC-30AD, an autosampler Sil-30AC, column oven CTO-20AC and diode array detector SPD-M20A. A reverse phase column ACE Excel 3 (C18, PFP, 100 × 2.1 mm; from ACE^®^ Advanced Chromatography Technologies Ltd., Aberdeen, Scotland) and pre-column (SecurityGuard ULTRA, C18; from Phenomenex, Torrance, CA, USA) were used at 40 °C for the separation of individual polyphenols. The flow rate of the mobile phase was 0.25 mL/min, and the injected sample size was 1.0 µL or 0.2 µL depending on the concentration of the sample. Mobile phases consisted of 1% formic acid in Milli-Q water (mobile phase A) and 1% formic acid in methanol (mobile phase B). Separation was carried out for 40 min. under the following conditions: Gradient 0–27 min, 15–80% B; 27–29 min, 80–95% B; 29–35 min, isocratic 90% B, and re-equilibration of the system with 15% B 8 min prior to the next injection. All samples were kept at 4 °C during the analysis. 

Individual phenolic compounds were identified by comparing the retention times, MS spectra, and parent and daughter ion masses with those of the standard compounds presented in Table 2. MS data acquisitions were performed on LCMS 8040 with the ESI source operating in both positive and negative modes. Nitrogen was used as the nebulizing gas (3 L/min) and drying gas (15 L/min). The heat block temperature was 400 °C and the desolvation line (DL) temperature was 250 °C.

All standards (gallic acid, pyrogallol, catechin, procyanidin B, chlorogenic acid, syringic acid, epicatechin, ferulic acid, piceatannol, quercetin-3-glucuronide, quercetin-3-galactoside, naringin, quercetin-3-glucoside, rutin, resveratrol, ɛ-viniferin, quercetin, naringenin, kaempferol, apigenin) and chemicals (formic acid, methanol) used were of analytical grade and purchased from Sigma (Steinheim, Germany) or from Cayman Chemical (Ann Arbor, MI, USA).

### 2.4. Statistical Analysis

The sum of polyphenols was calculated based on 12 major individual polyphenols detected in the shoots and canes of each grapevine cultivar. The obtained results were subjected to both, one-way and two-way ANOVA. In order to evaluate the effects of the cultivar on the content of polyphenolic compounds, the least significant differences were calculated by using Fisher’s Least Significant Difference (LSD) test and the significant differences (at *p* ≤ 0.05) were marked with different alphabetic letters (a, b, …). In order to evaluate the mean effects of variables, the results of the two-way ANOVA are presented with a significance level of * *p* ≤ 0.05, ** *p* ≤ 0.01 and *** *p* ≤ 0.001.

## 3. Results

### 3.1. The Sum of Polyphenols and Individual Polyphenolic Compounds in Shoots

Up to 19 different polyphenolic compounds were identified from the shoots and canes of grapevines, which were either in higher or lower concentrations. There were three flavanols (catechin, epicatechin, procyanidin B), five flavonols (quercetin, quercetin-3-glucoside + quercetin-3-galactoside, quercetin-3-glucuronide, kaempferol, rutin), six phenolic acids (ellagic acid, gallic acid, syringic acid, caffeic acid, chlorogenic acid, ferulic acid), three stilbenoids (resveratrol, ε-viniferin, piceatannol), and one flavanone (naringenin) and one flavone (apigenin). Still, many of these compounds were present in a very low concentration. Therefore, not all of the results are presented in this research article, but only the twelve most dominant ones. Further, the sum of polyphenols was the highest in cultivar Rondo (Table 3).

In the present experiment, the most abundant polyphenolic compounds determined from the grapevine shoots in descending order were quercetin-3-glucuronide > quercetin-3-glucoside + quercetin-3-galactoside > quercetin > rutin > kaempferol > procyanidin B > epicatechin > catechin > apigenin > ε-viniferin > naringenin for interspecific hybrid cultivars Zilga and Hasansky Sladky (see Table 3). Cultivar Rondo differed in the order for the identified compounds, and accordingly they were quercetin-3-glucuronide > quercetin-3-glucoside + quercetin-3-galactoside > rutin > quercetin > kaempferol > catechin > procyanidin B > apigenin > epicatechin > naringenin and ε-viniferin. For all three cultivars, quercetin-3-glucuronide was the most abundant, accounting for 74–87%. The shoots of cultivar Zilga had the highest content of epicatechin, procyanidin B, quercetin, apigenin and ε-viniferin in comparison to cultivars Hasansky Sladky and Rondo. The shoots of cultivar Rondo presented high concentrations of quercetin-3-glucoside + quercetin-3-galactoside, quercetin-3-glucuronide, naringenin, kaempferol and rutin, while cultivar Hasansky Sladky was modest for all the compounds compared to the other cultivars.

### 3.2. Individual Polyphenols, Flavonoids and Stilbenoids in Canes 

In canes, ε-viniferin had the highest proportion in both dormancy phases (Table 4). The most abundant polyphenolic compounds determined from grapevine canes of endo-dormancy were in descending order ε-viniferin > catechin > resveratrol > quercetin-3-glucuronide > quercetin > epicatechin > quercetin-3-glucoside + quercetin-3-galactoside > procyanidin B > kaempferol > naringenin in both cultivars. The order of the compounds started to differentiate according to cultivar and the contents of quercetin-3-glucoside + quercetin-3-galactoside, quercetin-3-glucuronide, kaempferol and quercetin were significantly lower in the phase of eco-dormancy. The results show that certain polyphenolic compounds such as catechin, epicatechin, procyanidin B from flavonoids group and ε-viniferin and resveratrol from stilbenoids group increased during eco-dormancy. At the same time quercetin-3-glucoside + quercetin-3-galactoside, quercetin-3-glucuronide, naringenin, kaempferol and quercetin dominated in endo-dormancy phase. The sum of individual polyphenols was higher in eco-dormancy phase in both cultivars, and the highest in cultivar Hasansky Sladky.

The mean effect of the cultivar was non-significant for catechin, quercetin-3-glucuronide and kaempferol, but the content of the other compounds depended on cultivar properties (Table 4). The dormancy phase of grape canes had significant effect on the content of individual polyphenols. Only the content of naringenin was one of the lowest detected in canes of eco-dormancy.

## 4. Discussion

In the present experiment, the major polyphenolic compounds determined from grapevine shoots differed for each interspecific hybrid cultivar. The differences can be attributed mainly to the diversity of *Vitis* species in cultivars’ pedigree. The content of polyphenols has shown great variability among inter- and intraspecies [25]. In our results, flavonols (quercetin-3-glucuronide, quercetin-3-glucoside + quercetin-3-galactoside, and quercetin) were the most abundant in shoots of all three cultivars. This agrees with Rusjan et al. [22] who also presented the flavonols as the major compounds in vines. In addition to flavonols, the shoots of Zilga had the highest content of flavanols (epicatechin, procyanidin B), similarly to cultivar Hasansky Sladky. However, the cultivar Rondo exhibited an increased level of kaempferol when compared to the two other cultivars. The least found were kaempferol from Zilga and Hasansky Sladky, and ε-viniferin from the shoot extracts of Rondo. In the present study, the overall effect of cultivar was non-significant for catechin, quercetin-3-glucuronide and kaempferol, but the content of the other compounds depended on cultivar properties. The significant effects of cultivar properties have also been reported previously [6,13,14,17,18]. Stems of cultivar Zilga have been described as a rich source of stilbenes by Pugajeva et al. [32] as well. The variability in the content of individual polyphenols may also relate to the cultivars’ earliness and growth start in spring. In spring the bud break of cultivars Zilga and Hasansky Sladky starts a week or two earlier when compared to Rondo, which means that the exposure of the two cultivars to late spring frosts and abiotic stress factors is increased. It has been demonstrated that the occurrence of stress factors at certain phenological stages induces various alterations in primary and secondary metabolism, thus affecting the content of biochemical compounds in grapevine [22,33]. 

In our results, the order of the compounds differentiated according to cultivar and the contents of flavonols were significantly lower in the phase of eco-dormancy. The results presented that flavanols (catechin, epicatechin, procyanidin B), and stilbenoids (ε-viniferin, resveratrol) increased in eco-dormancy, which is probably the result of temperature-induced metabolomic changes in grapevines. Abiotic stress factors have been shown to favor the accumulation polyphenols, especially stilbenoids in plants in relation to dormancy phases [27,33]. In our experiment, flavanols and a flavanone (naringenin) dominated in endo-dormancy phase. According to previous report, the most abundant compounds found in the *V. vinifera* grapevine canes are flavonols [5]. Overall, the dormancy phase of grape canes had significant effect on the content of individual polyphenols. Only the content of naringenin was one of the lowest detected in eco-dormant canes. Similar findings were presented by Eftekhari et al. [5], who referred that the potential of polyphenols in the samples varied according to vine growth and physiological stages, and naringenin was the lowest polyphenol detected in vegetative parts of *V. vinifera*. In our study, the grapevine leaf coloring and the first night frosts did not arrive until October 2019. The plants entered the endo-dormancy on time with the arrival of autumn weather conditions. The winter of 2019/2020 was up to 6.5 °C warmer than long-term mean and hence no winter damage occurred in canes, keeping also the abiotic stress factors low. Therefore, the properties of cultivar had the main influence in relation to grapevine phenology and length of the growth period of each individual cultivar. In our experiment, the canes of cultivar Zilga started maturation already in August 2019, while in Hasansky Sladky it begun at the end of September, which probably had significant impact on the accumulation of polyphenolic compounds. Similarly, Lachman et al. [33] concluded that interaction of important factors such as genotype and locality with its climatic conditions affect the formation of bioactive compounds, especially stilbenoids. The canes in endo-dormancy and eco-dormancy phases differed in the content of polyphenolic compounds. In canes, ε-viniferin from stilbenoids was the most abundant compound in both dormancy phases. In acknowledgement of our results, these bioactives have been declared as the main compounds found in canes of many different *Vitis* genotypes [5,6,13,25]. The accumulation of main stilbenoids (ε-viniferin, resveratrol) occur primarily in the woody parts of grapevine, followed by the roots, canes, and the stems [6,34].

## 5. Conclusions 

In conclusion, the flavonoid and stilbenoid contents in grapevine shoots and canes differed based on the cultivar properties and dormancy phase. In shoots, for all three cultivars, the most dominant compounds were flavonols (quercetin-3-glucuronide, quercetin-3-glucoside + quercetin-3-galactoside) and the least found were naringenin and ε-viniferin. In canes, the most abundant compounds in both dormancy phases were stilbenoids (especially ε-viniferin). The order of the compounds in woody canes differentiated according to cultivar. In the phase of endo-dormancy, increased amounts of flavonols and less stilbenoids were determined, while in eco-dormancy level of flavanols and stilbenoids increased. These results present high variability and potentiality of grapevine shoots and canes to be explored for the extraction of polyphenolic compounds. The vineyard pruning waste can be a valuable source of flavonoids and stilbenoids. Depending on the purpose of use and desired polyphenolic compounds, the selection of cultivar and different plant organs need to be considered correspondingly. Further research is required to explore the possible use of the obtained extracts to be used in food, pharmaceuticals, cosmetics industries or re-used for wine industry (e.g., similarly to oak chips).

## Figures and Tables

**Table 1:** Monthly mean, minimum, maximum temperatures and precipitation in 2019–2020, and average of 30 years (1991–2020) *.

Year	Month	Temperatures, °C				Precipitation, mm	
		Mean	Min	Max	Average of 30 Years	Monthly Sum	Average of 30 Years
2019	January	−5.7	−19.6	3.0	−4.1	50	48
	February	−0.2	−9.8	7.6	−4.4	43	39
	March	1.2	−10.6	11.8	−0.5	50	36
	April	8.1	−3.0	24.6	5.9	4	35
	May	11.6	−0.9	27.8	11.5	50	54
	June	18.8	6.3	30.1	15.5	70	88
	July	16.4	7.0	31.1	18.0	76	67
	August	16.8	6.5	26.6	16.7	58	79
	September	12.0	−0.6	25.8	11.8	83	55
	October	7.0	−4.4	15.0	6.0	87	68
	November	2.6	−7.6	11.2	1.2	73	55
	December	1.8	−5.6	9.0	−2.1	46	51
2020	January	2.4	−4.1	8.6	−4.1	27	39
	February	1.1	−7.6	9.0	−4.4	78	36
	March	2.5	−6.0	14.1	−0.5	30	35

* According to Estonian Weather Service observation data (Tartu-Tõravere climate normals, https://www.ilmateenistus.ee/kliima/kliimanormid/?lang=en (19 April 2021)).

**Table 2:** Retention times and MS transition list of analyzed phenolic compounds.

Compound	Retention Time (min)	Parent Ion [M + H]+	Parent Ion [M − H]−	Product Ion (*m*/*z*)
Gallic acid	2.33		169	125
Pyrogallol	2.34		125	79
Catechin	5.47	291		139
Procyanidin B	5.68	579		127
Chlorogenic acid	6.44	355		163
Syringic acid	8.25	199		140
Epicatechin	8.38	291		139
Ferulic acid	11.04	195		177
Piceatannol	12.84	245		107
Quercetin-3-glucuronide	14.08	479		303
Quercetin-3-galactoside	14.24	465		303
Naringin	14.36	581		273
Quercetin-3-glucoside	14.26	465		303
Rutin	14.15	611		303
Resveratrol	15.28	229		107
ɛ-Viniferin	18.31	455		107
Quercetin	19.10	303		153
Naringenin	19.25	273		153
Kaempferol	21.34	287		153
Apigenin	21.48	271		153

**Table 3:** The most abundant polyphenols (mg kg^−1^ d.w.) in grapevine shoots of three grapevine cultivars pruned in July 2019.

Polyphenolic Compounds (mg kg^−1^ dw)		Grapevine Cultivars		
		Zilga	Hasansky Sladky	Rondo
Flavanols	(+)-Catechin	29.2 ± 1.0 ^a^	14.9 ± 1.6 ^b^	30.2 ± 1.9 ^a^
	(−)-Epicatechin	40.0 ± 1.3 ^a^	21.9 ± 2.4 ^b^	5.2 ± 0.5 ^c^
	Procyanidin B	74.4 ± 2.8 ^a^	23.8 ± 2.8 ^b^	21.6 ± 3.5 ^b^
Flavonols	Quercetin-3-glucoside+ Quercetin-3-galactoside	807.9 ± 46.1 ^b^	456.8 ± 51.2 ^c^	1201.4 ± 80.1 ^a^
	Quercetin-3-glucuronide	4809.4 ± 283.9 ^b^	4782.8 ± 711.5 ^b^	7353.4 ± 579.7 ^a^
	Quercetin	420.4 ± 8.0 ^a^	98.1 ± 8.9 ^c^	192.0 ± 13.3 ^b^
	Kaempferol	74.4 ± 3.6 ^b^	35.4 ± 4.2 ^c^	116.4 ± 5.4 ^a^
	Rutin	193.8 ± 6.6 ^b^	75.5 ± 11.7 ^c^	517.3 ± 44.0 ^a^
Flavanones	Naringenin	3.2 ± 0.0 ^b^	2.0 ± 0.2 ^c^	4.9 ± 0.3 ^a^
Flavones	Apigenin	20.9 ± 0.6 ^a^	8.6 ± 1.2 ^b^	5.3 ± 0.2 ^c^
Stilbenoids	ε-viniferin	4.2 ± 0.5 ^a^	1.9 ± 0.2 ^c^	2.1 ± 0.2 ^b^
	Resveratrol	n.d.	n.d.	n.d.
Sum of individual polyphenols		6477.8 ± 341.6 ^b^	5521.5 ± 788.0 ^c^	9449.8 ± 727.7 ^a^

Different letters in the same row present statistically significant differences (effect of cultivar) at *p* ≤ 0.05. All data are expressed as mean ± S.D.; d.w., dry weight: n.d.—not detected.

**Table 4:** The most abundant polyphenols (mg kg^−1^ dw) in grapevine canes according to cultivars and the phase of dormancy in canes (endo-dormancy—October 2019; eco-dormancy—March 2020).

Polyphenols, mg kg^−1^ dw		Grapevine Cultivars					
		Zilga		Hasansky Sladky		Mean Effect of	
Compounds		Endo-	Eco-	Endo-	Eco-	cv	Dormancy Phase
		**Dormacy**	**Dormancy**	**Dormancy**	**Dormancy**		
Flavanols	(+)-Catechin	36.0 ± 5.3 ^b^	224.5 ± 35.3 ^a^	31.3 ± 1.7 ^b^	213.7 ± 21.5 ^a^	n.s.	***
	(−)-Epicatechin	6.1 ± 1.4 ^c^	61.8 ± 7.2 ^a^	2.7 ± 0.2 ^c^	37.2 ± 1.8 ^b^	***	***
	Procyanidin B	3.2 ± 1.4 ^c^	49.2 ± 8.6 ^a^	1.3 ± 0.3 ^c^	32.4 ± 3.2 ^b^	**	***
Flavonols	Quercetin-3-glucoside +Quercetin-3-galactoside	3.6 ± 1.6 ^b^	0.9 ± 0.1 ^c^	5.5 ± 0.4 ^a^	2.2 ± 0.2 ^c^	*	**
	Quercetin-3-glucuronide	16.7 ± 8.4 ^a^	1.5 ± 0.2 ^b^	11.9 ± 0.8 ^a^	2.1 ± 0.2 ^b^	n.s.	**
	Quercetin	9.5 ± 0.8 ^a^	1.3 ± 0.4 ^c^	7.2 ± 0.3 ^b^	0.1 ± 0.0 ^d^	***	***
	Kaempferol	2.4 ± 0.7 ^a^	0.1 ± 0.0 ^c^	1.6 ± 0.3 ^b^	0.1 ± 0.0 ^c^	n.s.	***
	Rutin	n.d.	n.d.	n.d.	n.d.	n.d.	n.d.
Flavanones	Naringenin	1.0 ± 0.1 ^b^	1.0 ± 0.1 ^b^	1.3 ± 0.0 ^a^	1.2 ± 0.1 ^a^	***	n.s.
Flavones	Apigenin	n.d.	n.d.	n.d.	n.d.	n.d.	n.d.
Stilbenoids	ε-viniferin	595.5 ± 69.6 ^c^	931.6 ± 72.8 ^a^	765.6 ± 87.4 ^b^	1042.8 ± 24.2 ^a^	**	***
	Resveratrol	21.4 ± 2.9 ^d^	44.0 ± 2.4 ^b^	32.4 ± 7.9 ^c^	186.9 ± 3.4 ^a^	***	***
Sum of individual polyphenols		696.2 ± 73.5 ^d^	1267.1 ± 108.8 ^b^	861.2 ± 94.9 ^c^	1518.9 ± 34.5 ^a^	*	***

Different letters in the same row present statistically significant differences at *p* ≤ 0.05. All data expressed as mean ± SD. The mean effects of cultivar and dormancy phase are presented as a significance level of * *p* ≤ 0.05, ** *p* ≤ 0.01 and *** *p* ≤ 0.001; n.s.—non-significant; n.d.—not detected.

## Data Availability

Data is contained within the article.
